# Chewing gum reduces visually induced motion sickness

**DOI:** 10.1007/s00221-021-06303-5

**Published:** 2022-01-07

**Authors:** Mara Kaufeld, Katharina De Coninck, Jennifer Schmidt, Heiko Hecht

**Affiliations:** 1 Human Systems Engineering (MMS), Fraunhofer Institute for Communication, Information Processing and Ergonomics (FKIE), Zanderstr. 5, 53111 Bonn, Germany; 2 Hochschule Döpfer University of Applied Sciences, Cologne, Germany; 3 Muenster School of Health, FH Muenster University of Applied Sciences, Münster, Germany; 4 Psychologisches Institut, Johannes Gutenberg-University Mainz, Mainz, Germany

**Keywords:** Visually induced motion sickness, Virtual reality, Simulator sickness, Chewing gum, Ginger

## Abstract

Visually induced motion sickness (VIMS) is a common side-effect of exposure to virtual reality (VR). Its unpleasant symptoms may limit the acceptance of VR technologies for training or clinical purposes. Mechanical stimulation of the mastoid and diverting attention to pleasant stimuli-like odors or music have been found to ameliorate VIMS. Chewing gum combines both in an easy-to-administer fashion and should thus be an effective countermeasure against VIMS. Our study investigated whether gustatory-motor stimulation by chewing gum leads to a reduction of VIMS symptoms. 77 subjects were assigned to three experimental groups (control, peppermint gum, and ginger gum) and completed a 15-min virtual helicopter flight, using a VR head-mounted display. Before and after VR exposure, we assessed VIMS with the Simulator Sickness Questionnaire (SSQ), and during the virtual flight once every minute with the Fast Motion Sickness Scale (FMS). Chewing gum (peppermint gum: *M* = 2.44, SD = 2.67; ginger gum: *M* = 2.57, SD = 3.30) reduced the peak FMS scores by 2.05 (SE = 0.76) points as compared with the control group (*M* = 4.56, SD = 3.52), *p* < 0.01, *d* = 0.65. Additionally, taste ratings correlated slightly negatively with both the SSQ and the peak FMS scores, suggesting that pleasant taste of the chewing gum is associated with less VIMS. Thus, chewing gum may be useful as an affordable, accepted, and easy-to-access way to mitigate VIMS in numerous applications like education or training. Possible mechanisms behind the effect are discussed.

**Supplementary Information:**

The online version contains supplementary material available at 10.1007/s00221-021-06303-5.

## Introduction

Virtual reality (VR) technologies and the use of head-mounted displays (HMD) are growing in popularity for a variety of applications, including entertainment, education, and emergency response training (see, e.g., Ahir et al. 2020; Caserman et al. 2018; Grabowski and Jankowski 2015; Hartmann and Fox 2020; Kinateder et al. 2014). Unfortunately, many users experience mild or severe motion sickness symptoms, such as nausea, disorientation, or oculomotor difficulties (Kennedy et al. 1993; Moss and Muth 2011). If these symptoms are not triggered by physical motion alone, but rather involve visual stimuli at odds with the other senses, this malaise is referred to as visually induced motion sickness (VIMS) (for an overview, see Bronstein et al. 2020; Caserman et al. 2021; Golding and Gresty 2015; Keshavarz et al. 2014) or as cybersickness. The Bárány Society also developed more specific diagnostic criteria for motion sickness and VIMS regarding various adverse reactions and their occurrence, duration, and remission (see Cha et al. 2021).

Medical countermeasures include drugs, such as antihistamines and anticholinergics, which are effective in reducing motion sickness, but unfortunately also cause serious side-effects such as drowsiness, lethargy, and dry mouth (Koch et al. 2018; Shupak and Gordon 2006). The most successful behavioral countermeasure is adaptation to the nausea-inducing stimuli through prolonged exposure (Heutink et al. 2019; Jannu 2015; Young et al. 2003). Although adaptation is very effective, it can be time-consuming and inconvenient or deemed not acceptable by the user in therapeutic use settings.

To overcome practical limitations in modern VR technologies due to VIMS, innovative and easy-to-administer behavioral countermeasures are needed. A study by Bos (2015) showed that head vibration reduced the amount of sickness by 25% and mental distraction by 19%, with a combined effect of both amounting to 39% reduction. Other studies have shown that mechanical stimulation of the mastoid region (Weech et al. 2018), pleasant odors (Keshavarz et al. 2015), and diverting attention to pleasant musical stimuli can ameliorate VIMS (Keshavarz and Hecht 2014). It is likely that chewing gum also has a positive impact, since it may exercise a positive effect by indirect mastoid stimulation effected through chewing, and at the same time by pleasurable flavor experience. In the following, we describe the sensory conflict theory and provide a model in which the possible mechanisms of gum chewing on VIMS are embedded.

**Fig. 1:**
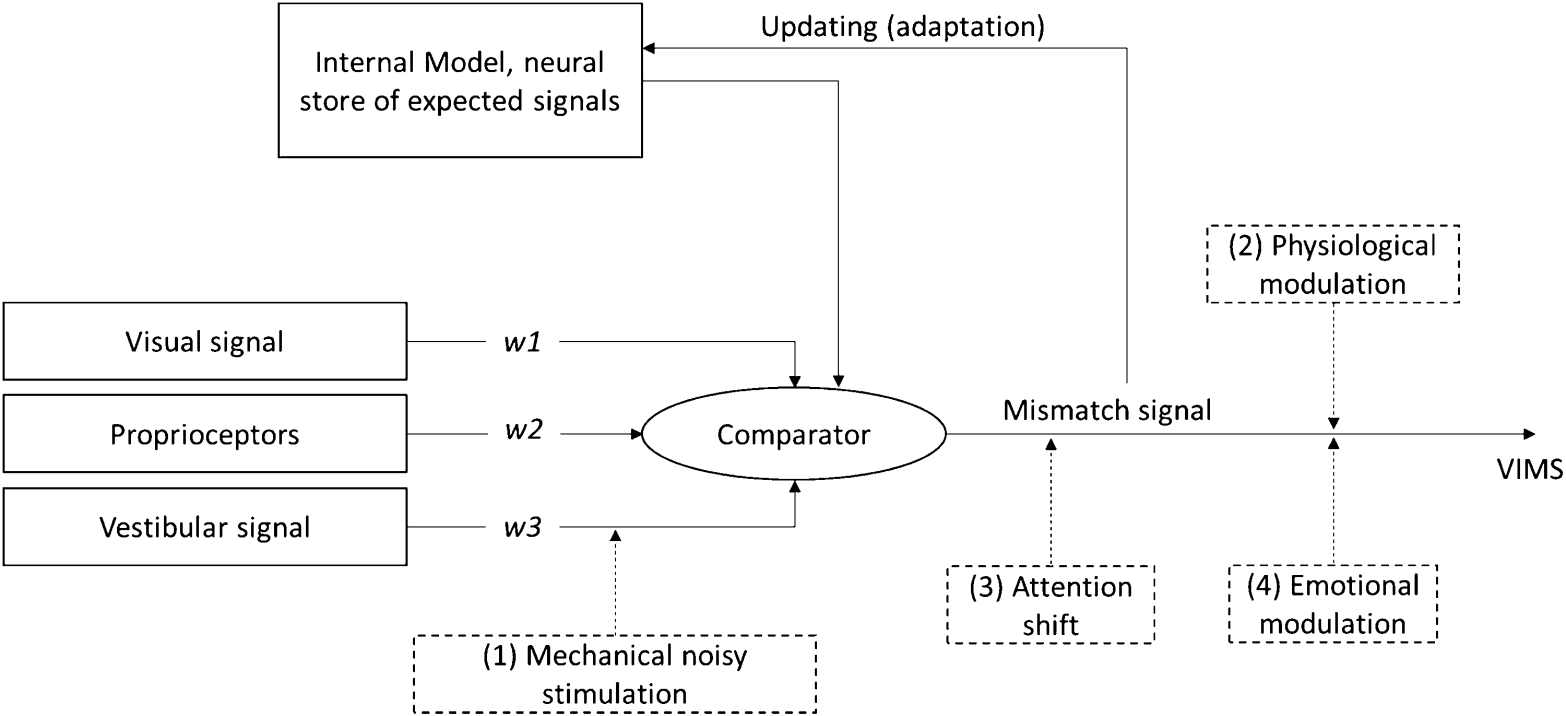
Illustration how chewing gum could modulate the occurrence of VIMS based on the sensory conflict theory. Modified from Keshavarz et al. (2014). Possible mechanisms how chewing gum might act are depicted in dashed lines (1)–(4). w1–w3 represent the weighting of the different afferent sensory inputs. Note that the mechanisms may work in isolation or in unison

According to the sensory conflict theory of motion sickness (Reason 1978; Reason and Brand 1975)*,* sensory mismatches between visual, vestibular, and proprioceptive information lead to unpleasant symptoms (see Fig. 1). Moreover, the authors suggested that the conflict is not just lying in incompatible sensory inputs but also in deviations from past and present sensory information. In the model displayed in Fig. 1, we illustrate four mechanisms how gum chewing might modulate the emergence of VIMS in the context of sensory conflicts.

(1) The mechanical process of chewing could stimulate the mastoid region, thus adding noise to the vestibular afferences and down-weighting the visual–vestibular conflict. (2) Ingredients of chewing gums, such as ginger, could have an effect on physiological processes like arousal or hormones. (3) Chewing gum could shift attention away from the provocative stimulus. (4) Chewing gum could evoke a positive emotional state. Note that, according to the model, the comparator generates a mismatch signal not only if one sensory input is at odds with another but also, if a congruent sensory input does not match the expected sensory input based on prior experience.

In the following, we take a closer look at these four potential mechanisms described in Fig. 1 to gauge their potential importance based on the existing literature.

Stimulating the vestibular system (1) to reduce the visual-vestibular conflict in VIMS-provoking simulations has been investigated using galvanic vestibular stimulation. The purpose of this technique typically is to modify vestibular input through galvanic signals from electrodes placed close to the mastoid (Curthoys and Macdougall 2012; Day and Fitzpatrick 2005; Swaak and Oosterveld 1975). It has been shown that synchronized as well as noisy galvanic stimulation of vestibular nerves can lead to a reduction of VIMS (Cevette et al. 2012; Gálvez-García et al. 2015; Reed-Jones et al. 2007; Sra et al. 2019; Weech et al. 2020) but caused minor feelings of discomfort, like itching and tingling, in healthy individuals (Utz et al. 2011). Weech et al. (2018) investigated the effect of noisy bone-conducted vestibular stimulation on VIMS, showing that noisy mechanical stimulation of the vestibular system can likewise reduce VIMS. The above-mentioned study by Bos (2015) had used vibrations administered through a headrest, which were equally passive. If actively produced vibrations are comparable, we would expect that the mechanical process of chewing gum stimulates the vestibular system in a noisy manner, thereby down-weighting the vestibular afferents, and reducing the visual–vestibular conflict when exposed to nauseating stimuli. Previous studies showed that chewing could be measured with bone vibration sensors placed close to the mastoid (Van der Bilt et al. 2010; Zhang and Amft 2016). The association of chewing and postural stability has been demonstrated in the way that gum chewing improved postural stability when standing upright on an unstable surface without visual input (Alghadir et al. 2015). However, the mechanism by which chewing gum improved postural stability remains unclear.

Besides the mechanical effects of chewing gum, it can also affect motion sickness through active ingredients (2). Medicated chewing gums were shown to be an effective drug delivery system in many application fields such as pain relief, prevention of dental caries, vitamin or mineral supplementation, and travel sickness (Jain et al. 2019; Khatun and Sutradhar 2012; Kumari et al. 2020). Chewing releases active substances from the gum, which are systemically distributed after absorption through the oral mucosa. Most commercially available chewing gums against motion sickness contain the antihistamine dimenhydrinate (Jacobsen et al. 2004; Skofitsch and Lembeck 1983). Since dimenhydrinate leads to side-effects, safer agents should be used against motion sickness. Jarisch et al. (2014) found that vitamin C was effective in suppressing symptoms of seasickness on a life raft. Ginger root is another remedy against motion sickness with fewer undesirable side-effects, as compared to conventional drug agents (Pongrojpaw et al. 2007). Some evidence exists for its effectiveness in reducing general motion sickness (Grøntved et al. 1988; Mowrey and Clayson 1982), nausea (Pongrojpaw et al. 2007), and VIMS (Lien et al. 2003). The mechanism of action appears to be that ginger suppresses the increase in plasma vasopressin levels, thereby alleviating gastric dysrhythmias and nausea (Kim et al. 1997; Lien et al. 2003). However, other studies found no ameliorative effect of ginger on motion sickness (Schartmüller and Riener 2020; Stewart et al. 1991). Notably, the mentioned studies differed greatly in terms of the ginger dosage, its administration, and the dependent measures investigated. Overall, the effects of ginger are contradictory and need to be further explored with regard to VIMS (for an overview, see Palatty et al. 2013).

Distraction (3) may also be involved in the sense that the motor task of gum chewing diverts attention away from nausea-inducing stimuli, although the mental effort involved in gum chewing is likely very small. Note that the role of distraction in the genesis of VIMS is far from clear. There is both evidence for a positive effect of mental distraction (Bos 2015) as well as counter-evidence (Yen Pik Sang et al. 2003).

Some evidence exits that emotional modulation (4) by pleasant distractors may play a role in the genesis of VIMS. For instance, pleasant music, odors, and airflow were shown to reduce VIMS (D'Amour et al. 2017; Keshavarz et al. 2015; Keshavarz and Hecht 2014; Peck et al. 2020; Ranasinghe et al. 2020). We assume that a reduction in VIMS occurred in the mentioned studies, because the pleasant stimuli evoked a pleasant emotional state or a positive mood, which distracted the subjects or diverted their attention from the nauseating stimuli to the more pleasant stimuli. In the present study, taste could serve as such a pleasant distractor, since taste perception is processed via the limbic system and the hypothalamus, areas associated with emotions (Yamamoto 2008). Additionally, negative emotions as well as poor emotion regulation are associated with greater nausea in chemotherapy patients (Ashkhaneh et al. 2015; Olver et al. 2014). Furthermore, perceived pleasant tastes of administered drinks were shown to predict nausea in subjects exposed to an optokinetic drum (Williamson et al. 2005).

Some evidence exists that the effect of chewing peppermint gum can be comparable to that of an anti-emetic drug (4 mg ondansetron) for postoperative nausea (Darvall et al. 2017). The willingness to try chewing gum against postoperative nausea and vomiting was found to be very high (84%), especially among younger patients (95%) (Darvall et al. 2019).

In the present study, we investigated chewing gum as a potential countermeasure to VIMS. To our knowledge, no internationally published study concerning chewing gum as countermeasure for VIMS exists. The aim of the present study was to examine the impact of chewing gum and pleasant taste on VIMS. Due to its familiarity and a lack of side-effects in contrast to other countermeasures, chewing gum might be an effective and highly accepted counteragent to VIMS in VR. We exposed subjects to a 15-min helicopter VR simulation, while they chewed either a peppermint chewing gum, a ginger chewing gum, or no chewing gum in the control group. We collected ratings of VIMS and pleasantness of taste to investigate the impact of chewing gum.

Taken together, the literature shows evidence that VIMS can be alleviated by noisy mechanical or galvanic stimulation of the mastoid region, using ginger as an active ingredient, and by pleasant distractors. Therefore, we assume that subjects will experience less VIMS symptoms when chewing gum as compared to not chewing gum. Due to the known ameliorative effect of pleasant odors on VIMS along with the previously reported beneficial effects of pleasant taste on nausea, we assume that the more pleasant the taste of the chewing gum is subjectively perceived, the less VIMS is reported.

If both the peppermint and the ginger chewing gum appear pleasant and affect VIMS in a similar manner, vestibular stimulation (1), attention shift (3), and emotional modulation (4) could all be responsible for VIMS reduction. However, if the effects of both chewing gums on VIMS are equal, but one flavor is more attractive, then (4) can be ruled out. In turn, if the ginger gum is more effective in reducing VIMS compared to the peppermint gum, the effect would rather be attributable to physiological modulation (2) by the active ingredient. Thus, depending on the outcome, we can narrow down the potential mechanisms.

## Method

### Study design

We conducted experimental sessions between June and July 2020. Before we started collecting the data, we pre-registered the research protocol on 5/19/2020 on the website https://aspredicted.org/ with the number #41329. Before participation, subjects were informed that they were participating in a VR helicopter study investigating the prevention of VIMS. However, we did not inform them about the type of prevention investigated in the study until the very end of the experiment. As compensation, the subjects were offered the opportunity to participate in a raffle (three 20 Euro vouchers for different shops) or to receive course credit. They were also informed that participation was voluntary and that they could decide to discontinue the study at any time without giving a reason and without consequences. As an additional precaution, we chose a rating above 15 on the Fast Motion Sickness Scale (FMS) as a cut-off value to abort the experiment. All subjects included in the study signed an informed consent. The research protocol was approved by the institutional ethics board (HSD Hochschule Döpfer University of Applied Sciences) and was conducted in accordance with the declaration of Helsinki.

We assigned the subjects into three groups (control, peppermint, or ginger group) using a stratified randomization approach. During the assignment process, we stratified for gender and age as these may have an influence on VIMS (see, e.g., Keshavarz et al. 2018; Shafer et al. 2017).

In a between-subjects design, subjects completed a 15-min VR helicopter flight in the position of a crewmember, either without chewing gum, chewing a peppermint-flavored gum, or chewing a ginger-flavored gum. For some analyses, we also included time (pre–post) or the whole time course as within-subjects factor to examine the interaction between time and group. We assessed VIMS as our dependent variable with self-report measures.

### Measures

#### VIMS measures

All questionnaires were created with a survey tool (LimeSurvey) and filled in on a tablet in an offline version. VIMS was measured twice, before and after exposure, using the Simulator Sickness Questionnaire (SSQ) (Kennedy et al. 1993), and every minute during simulation using the FMS (Keshavarz and Hecht 2011). The SSQ contains 16 symptoms (i.e., headache, nausea, and eyestrain) rated on 4-point Likert scales with the choice of selecting none (0), slight (1), moderate (2), or severe (3). Values were weighted and summed for the total score and the subscales nausea, oculomotor distress, and disorientation according to the instructions of Kennedy et al. (1993). A study by Bouchard et al. (2007) found Cronbach's alpha to be 0.87. We applied the SSQ before and after VR exposure to ensure that the groups did not differ in baseline scores.

Additionally, we used the FMS (Keshavarz and Hecht 2011) as a single-item scale to continuously monitor VIMS symptoms every minute of VR exposure. The scale ranges from 0 (no sickness at all) to 20 (frank nausea). Peak FMS scores had been highly correlated with the SSQ subscales nausea (*r* = 0.83), disorientation (*r* = 0.80), oculomotor (*r* = 0.61), and the total score (*r* = 0.79) (Keshavarz and Hecht 2011). We used the FMS in addition to the SSQ, because it provides a broader range of response options and the ability to assess VIMS during exposure. According to the FMS instructions, we asked subjects to focus on general discomfort, nausea, and stomach discomfort, and to ignore other feelings such as excitement, fatigue, boredom, and nervousness.

In addition, we used the Motion Sickness Susceptibility Questionnaire (MSSQ) to ensure that the groups did not differ at baseline in terms of participant’s individual susceptibility to motion sickness (Golding 2006). The short form of the MSSQ, which was applied in our study, asks for the previous sickness occurrences in cars, buses, trains, aircrafts, small boats, large ships, swings, carousels in playgrounds, and leisure park attractions. Subjects can rate their experiences by selecting from not applicable/never traveled (coded with t), never felt sick (0), rarely felt sick (1), sometimes felt sick (2), and frequently felt sick (3). It asks separately for childhood experiences before the age of 12 and the experiences over the last 10 years. Calculation of the total scores followed the instructions of Golding (2006). In a validation study of the MSSQ, predictive validity for motion sickness showed a median of *r* = 0.51. Cronbach's alpha was 0.87 and the test–retest reliability was around *r* = 0.90 (Golding 2006).

#### Other measures

The experimental groups were asked additional questions about the taste and duration of the chewing gum flavor. A custom bipolar item was used to ask how pleasant the taste of the chewing gum was perceived, ranging from very unpleasant (1) to very pleasant (6). Subjects were instructed to pay attention only to the taste of the chewing gum and not to its consistency. Furthermore, we asked the subjects how long they perceived the taste of the chewing gum to last during the simulation (not at all, only at the beginning of the simulation, until the middle of the simulation, close to the end of the simulation).

In addition to the aforementioned questionnaires, after the experiment, we also collected questionnaire data for another research project and administered the Multidimensional Assessment of Interoceptive Awareness (MAIA) (Mehling et al. 2012), Somatic-Symptom-Scale 8 (SSS-8) (Gierk et al. 2014; German version: Löwe and Voigt 2015), Measure of technology commitment (Neyer et al. 2012), and Igroup Presence Questionnaire (IPQ) (Schubert 2003).

### Participants

According to a-priori power analysis with G-Power version 3.1.9.2 (Faul et al. 2009), a sample size of *n* = 74 would be sufficient (with alpha = 0.05, power = 0.80) to detect effect sizes (*η*_p_^2^ = 0.099 corresponds to Cohen’s *f* = 0.33) similar to those reported by Keshavarz et al. (2015) for the interaction of time and odor, using the FMS (Keshavarz and Hecht 2011). Anticipating some dropouts and early aborts, we recruited 90 participants using an email list of the HSD Hochschule Döpfer University of Applied Sciences and social media. The participants were assigned to one of the three groups (control, peppermint, and ginger).

Exclusion criteria were known health issues like damages of the vestibular organs as well as diseases of the eyes that restrict vision and cannot be corrected-to-normal vision (e.g., through glasses or contact lenses). A necessary precondition was a normal or a corrected to normal vision, which was tested beforehand with an EN ISO 8596/7 vision chart. With regard to the chewing gum and its ingredients, we screened for fructose and/or sorbitol intolerance. At the time of the study or before, no motion sickness medications should have been consumed. In addition, extreme fear of heights was an exclusion criterion, as we used a helicopter simulation to induce VIMS.

### Apparatus and stimuli

During the experiment, the subjects were seated on a stationary chair without armrests, had a presenter remote control in their hands, and wore a VR-HMD via which the virtual helicopter flight was displayed. The simulation was implemented using VBS 3 (Bohemia Interactive Simulations, n.d.), an environment for generating virtual 3D trainings for emergency personnel. The PC we used contained an Xeon CPU E5-1620 0 (3.60 GHz) processor (Intel, Santa Clara, United States), 32 GB (DDR3) of RAM and a GeForce GTX 1080 graphics card (with 8 GB GDDR5X memory) (Nvidia, Santa Clara, United States). The operating system was Windows 10 Pro (version 10.0.18363). As VR-HMD, we used the Vive Cosmos (HTC, Taoyuan), which offers a resolution of 1440 × 1700 pixels per eye with a 90 Hz refresh-rate, a diagonal field of view of 110° (HTC, n.d.), and a mechanism for adjusting the interpupillary distance (IPD). Due to the flip-up visor, the Vive Cosmos can be worn with glasses. Helicopter sound was delivered via integrated on-ear headphones and the volume was set to 70 in the windows settings during the VR exposure.

**Fig. 2:**
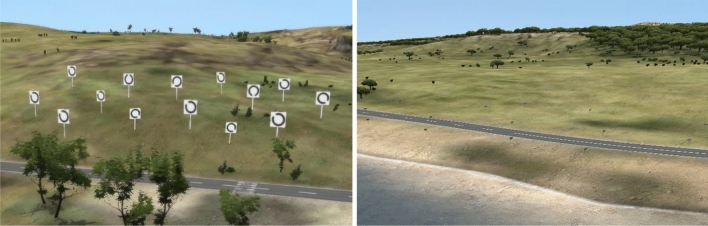
Screenshots of the simulation. Left panel: visual search task with Landolt rings. Right panel: landscape seen during the breaks

We created a simulation inspired by a search and rescue training for helicopter crews, where the crew scans the landscape for injured or missing people. The subject was flown in a helicopter using the autopilot mode along a fixed route around the coast of a peninsula. Waypoints were set to implement the route, so that the helicopter flew the same route along the waypoints for each subject. To complete a visual search task, they looked out of the right rear door of the helicopter and scanned the landscape for signs with Landolt rings on them. The task was to press a "yes" button on a presentation remote control when they recognized a Landolt ring with an opening to the top in a set of 14 Landolt rings, and to press a "no" button when they did not (see Fig. 2).

We used this task to ensure that subjects were looking out of the helicopter and not into it, which would generate no or insufficient VIMS. The entire simulation contained 30 trials, each with 14 Landolt ring signs embedded in the environment, which were visible for 6 s. Subjects completed ten consecutive trials and, after a break, the next ten. During the break, the helicopter continued to fly through the landscape and the subjects did not know when the next set of Landolt rings would appear (see Fig. 2). To enable the subjects to recognize the Landolt rings, the helicopter flew slower during the trials and faster during the breaks. This resulted in a mixture of smooth and shaky movements during the flight. We conducted a preliminary study with seven subjects (*M*_age_ = 28.43 years; SD = 3.64) to test whether the simulation evoked sufficient VIMS. We found that this was the case with an FMS mean peak score of 6.29 (SD = 5.02).

### Selected chewing gums

To select chewing gums for the present study, we compared three chewing gums in a pre-test with 11 subjects (*M*_age_ = 26.89 years; SD = 3.45): a supposedly neutral mastic chewing gum, a peppermint chewing gum, and a ginger chewing gum. The subjects rated the mastic gum as unpleasant, not neutral in taste, and much tougher in consistency than the other chewing gums. For this reason, and also because commercial chewing gums are usually flavored, we decided to include only a peppermint and a ginger chewing gum of the brand Simply Gum (New York City, United States) in our study. These chewing gums are plastic-free, biodegradable, without synthetic content or added sweetener (Simply Gum, n.d.). According to the package information, the chewing gums contained real peppermint and ginger essential oils, respectively. The amount of peppermint and ginger oil could not be determined from the website or the packaging. For the study, the chewing gums were removed from the original packaging and repackaged in neutral brown packets, so that the type of gum was not identifiable. For identification, the packages were marked with a code. Our pre-test confirmed that the chewing gums were similar in terms of consistency and volume.

### Procedure

Prior to the experiment, subjects were informed about the procedure and about the possibility to terminate the study at any time without consequences. All subjects signed a written informed consent, successfully passed a vision test, and completed a demographic questionnaire, the pre-SSQ, and the MSSQ. Subsequently, the experimenter explained the tasks to be performed during VR exposure, presented the FMS in written form, and then handed out the chewing gum. Subjects were asked to place it in their mouth and chew it throughout the simulation. Then, they donned the VR-HMD and began the virtual helicopter flight in the position of a crewmember. Right at the beginning, they were told that they could adjust the IPD using the wheel on the side of the VR-HMD if the image was not sharp. During the 15-min virtual helicopter flight, subjects were exposed to sickness-inducing visual motion and completed the visual search task (Landolt rings). Meanwhile, they were asked to verbally rate their sickness every minute, using the single FMS item. The experimenter visually checked whether subjects chewed the gum and reminded them to do so when necessary. They then completed the post-SSQ and the questions on how pleasant and how long-lasting they perceived the taste. Finally, subjects were debriefed and left the laboratory once symptoms experienced during the experiment had subsided. Note that you can find information on COVID-19 precautions in the supplementary materials.

### Statistical analysis and design

All statistical analyses were performed using SPSS (version 25) or JASP (version 0.13.1, 0.14). The a-priori significance level was set to *p* < 0.05. Although most of our data were not normally distributed (Shapiro–Wilk. *p* < 0.05), we chose parametric over nonparametric tests, because ANOVAs were shown to be relatively robust against violations of the normality assumption (see, e.g., Blanca et al. 2017). We conducted a mixed 2 × 3 ANOVA including the within-subject-factors time (pre–post) and the between-subjects-factor group (peppermint gum, ginger gum, and no gum) for the SSQ data. For the FMS data, we calculated a between-subjects-ANOVA with the factor group using FMS peak scores, i.e., the highest score a subject reported during VR exposure. Additionally, we performed a mixed 16 × 3 ANOVA to analyze the interaction of FMS time course and group. It included all 16 FMS scores (within-subjects factor time course), and group as between-subjects factor. For post hoc analyses, we used Helmert contrasts, comparing the control group against the two chewing gum groups and the peppermint and ginger groups against each other. Finally, we performed a one-tailed correlation analysis for pleasant taste and VIMS (SSQ, FMS peak scores) using the Spearman Brown Formula, since the collected data are not normally distributed.

After collecting all data, we excluded six subjects from the data set because of extremely high SSQ total pre-scores, which were identified as outliers in an SPSS boxplot analysis (1.5 interquartile ranges from median, which were SSQ scores of 44.88 or higher). After the experiment, the subjects were asked to comment on their experience and to state whether they had followed all instructions. We noted all reasons for non-compliance. Subsequently, two independent reviewers (without access to the data) judged the severity of the bias and decided which subjects were to be excluded from the analysis. Here, three subjects admitted to have made false statements regarding pre- and post-SSQ, two subjects had complaints due to excessive heat generated by the VR-HMD, one subject indicated reactance and a resistance to comply because of the military setting of the VR simulation, and one subject did not chew the gum. Thus, these seven subjects were excluded from the data analyses. According to our a-priori power analysis, a sample size of 74 would be sufficient. Thus, with 77 subjects remaining in our sample, the power is still adequate.

## Results

### Sample characteristics and baseline differences

The 77 subjects (43 female, 34 male) with a mean age of 34.01 years (SD = 14.15) included in the analyses were distributed among the three groups control (*n* = 27), peppermint (*n* = 27), and ginger (*n* = 23) as displayed in Table 1.

**Table 1:** Sample description divided by groups for age, gender, and Motion Sickness Susceptibility Questionnaire

	Control	Peppermint	Ginger	Total
N	27	27	23	77
Age	34.56 (13.56)	36.26 (15.81)	30.74 (12.60)	34.01 (14.15)
Sex	15 f, 12 m	14 f, 13 m	14 f, 9 m	43 f, 34 m
MSSQ	6.34 (6.87)	7.74 (8.84)	9.79 (7.99)	7.86 (7.82)

For age and MSSQ, values indicate *M* (SD)

*MSSQ* Motion Sickness Susceptibility Questionnaire

We found no significant differences among the groups with regard to age [*F*(2,74) = 0.98, *p* = 0.382], gender [*χ*^2^(2) = 0.41, *p* = 0.814], and MSSQ scores [*F*(2,74) = 1.22, *p* = 0.301]. Likewise, they did not differ in initial motion sickness (see Table 1). A one-way ANOVA on the pre-SSQ scores with group as between-subjects factor revealed no significant differences between the groups for the subscales nausea, *F*(2, 74) = 1.69, *p* = 0.191, oculomotor, *F*(2, 74) = 0.02, *p* = 0.978, disorientation, *F*(2, 74) = 0.32, *p* = 0.724, and the SSQ total score prior to VR exposure *F*(2, 74) = 0.23, *p* = 0.794 (see Table 2). This indicates that the groups did not differ on VIMS-related symptoms prior to exposure.

**Table 2:** Mean (SD) SSQ-pre and -post scores and mean (SD) peak FMS scores

VIMS measure		Gum			Total
		Control	Peppermint	Ginger	
Pre-SSQ	Nausea	7.77 (9.55)	12.37 (12.07)	7.88 (8.94)	9.42 (10.44)
	Oculomotor	10.95 (10.37)	11.51 (12.14)	11.53 (11.62)	11.32 (11.24)
	Disorientation	8.76 (11.02)	6.70 (11.17)	6.66 (10.17)	7.41 (10.73)
	Total	10.80 (9.86)	12.33 (10.87)	10.57 (9.48)	11.27 (10.02)
Post-SSQ	Nausea	26.50 (26.50)	22.97 (22.65)	17.00 (15.48)	22.43 (22.36)
	Oculomotor	22.46 (21.07)	14.88 (13.54)	13.84 (17.65)	17.23 (17.89)
	Disorientation	36.60 (35.20)	21.14 (24.83)	16.34 (19.53)	25.13 (28.66)
	Total	31.31 (27.93)	22.02 (19.08)	17.89 (17.28)	24.04 (22.57)
Peak FMS		4.56 (3.52)	2.44 (2.67)	2.57 (3.30)	3.22 (3.29)

*SSQ* Simulator Sickness Questionnaire, *FMS* Fast Motion Sickness Scale

### Manipulation check

All 77 subjects reported that they had not closed their eyes for prolonged periods during the simulation and clicked the buttons on the presentation remote control when the Landolt rings appeared. Performance data were available for 74 of the 77 subjects. The data of three subjects were missing due to technical recording failure. Of 30 trials, on average, 24.76 (SD = 3.68) were correctly identified (83%), 4.12 (SD = 2.62) were incorrectly identified (13%), and 1.12 (SD = 1.98) were missed altogether (4%). Due to the low number of missed trials, we assume that the subjects performed the tasks as instructed and therefore were exposed to the VIMS-inducing simulation.

### Chewing gum

The mixed ANOVA on the SSQ scores yielded significant main effects of time (pre–post) for all subscales [Nausea: *F*(1, 74) = 27.16, *p* < 0.001, *η*_p_^2^ = 0.27, Oculomotor: *F*(1, 74) = 9.98, *p* = 0.002, *η*_p_^2^ = 0.12, Disorientation: *F*(1, 74) = 28.49, *p* < 0.001, *η*_p_^2^ = 0.28], and the total score (*F*(1, 74) = 27.54, *p* < 0.001, *η*_p_^2^ = 0.27), indicating that SSQ scores were higher after VR exposure than before (see Table 1). All means and standard deviations for the VIMS measures are shown in Table 2.

Furthermore, a significant main effect of group was only found for the subscale disorientation, *F*(2, 74) = 3.72, *p* = 0.029, *η*_p_^2^ = 0.09, indicating that the chewing gum groups suffered less disorientation by 9.98 points (SE = 3.7), *p* = 0.009, *d* = 0.79 as compared to the control group. There was no significant difference between the peppermint and ginger group (*p* = 0.583). We found no other main effects for group, regarding the nausea, *F*(2, 74) = 1.07, *p* = 0.348, *η*_p_^2^ = 0.03., and oculomotor subscales, *F*(2,74) = 0.78, *p* = 0.460, *η*_p_^2^ = 0.02, or for the total score, *F*(2, 74) = 1.57, *p* = 0.216, *η*_p_^2^ = 0.04. We detected a non-significant trend for an interaction of time (pre-post) and group for the subscales oculomotor, *F*(2, 74) = 2.62, *p* = 0.080, *η*_p_^2^ = 0.07, disorientation, *F*(2, 74) = 2.82, *p* = 0.066, *η*_p_^2^ = 0.07, and the SSQ total score (see), *F*(2, 74) = 2.94, *p* = 0.059, *η*_p_^2^ = 0.07, but not for the subscale nausea *F*(2, 74) = 1.50, *p* = 0.231, *η*_p_^2^ = 0.04.

**Fig. 3:**
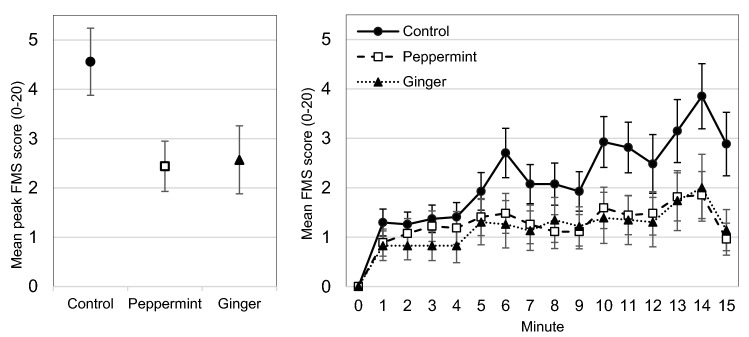
Mean peak FMS score separated by group (left) and time course of the FMS scores minute by minute separated by group (right). Error bars represent standard error of the mean. *FMS* Fast Motion Sickness Scale

A between-subjects ANOVA for the FMS mean peak scores revealed a main effect of group, *F*(2, 74) = 3.68, *p* = 0.03, *η*_p_^2^ = 0.09, indicating lower FMS mean peak scores for the chewing gum groups compared to the control group with a difference of 2.05 (SE = 0.76), *p* = 0.009, *d* = 0.65 (see Fig. 3). There was no statistical difference of the FMS mean peak scores between the peppermint and the ginger group (*p* = 0.894).

Figure 3 shows the complete time course of the FMS ratings. The mixed ANOVA that included each time point revealed a significant main effect for the time course, Huynh–Feldt corrected *F*(4.23, 312.64) = 20.01, *p* < 0.001, *η*_p_^2^ = 0.21, indicating increasing FMS scores over the time of VR exposure. We did not find a significant main effect of group, *F*(2, 74) = 2.29, *p* = 0.109, *η*_p_^2^ = 0.06, but a significant interaction of time course and group, Huynh Feldt corrected *F*(8.45, 312.64) = 2.30, *p* = 0.019, *η*_p_^2^ = 0.06, indicating diverging lines. Helmert contrasts showed a significant difference between the chewing gum groups and the control group of 0.97 (SE = 0.45), *p* = 0.035, *d* = 0.10 but no significant differences between the peppermint and the ginger group (*p* = 0.937) in the FMS time course.

### Pleasant taste

The taste ratings did not significantly differ between the peppermint (*M* = 4.26, SD = 1.43) and the ginger chewing gums (*M* = 3.83, SD = 1.64), *F*(1, 48) = 0.99, *p* = 0.324, *η*_p_^2^ = 0.02, and indicate that subjects perceived both chewing gums as rather pleasant. In the peppermint group, 17 out of 27 subjects correctly identified the flavor of the chewing gum (mint, peppermint, and menthol). In the ginger group, out of 23 subjects, only five correctly identified the flavor as ginger.

For correlation analysis, both chewing gum groups were included in this analysis. Pleasant taste correlated negatively with VIMS, *r*(48) = − 0.24, *p* = 0.050,^1^ as measured by the SSQ total score, but was uncorrelated with the FMS peak scores, *r*(48) = − 0.14, *p* = 0.159. However, when we asked subjects how long they perceived the taste of the chewing gum, four subjects stated that they did not perceive the taste at all during the simulation. We excluded these subjects from the analysis and recalculated the analysis using the modified data set. We then detected significant negative correlations for both the SSQ total score, *r*(44) = − 0.31, *p* = 0.020 and FMS mean peak score, *r*(44) = − 0.27, *p* = 0.037, suggesting that when pleasant taste is perceived, it is associated with less VIMS.

## Discussion

The aim of the present study was to investigate the impact of chewing gum and pleasant taste on VIMS. Our results showed that chewing flavored gum was effective in reducing VIMS symptoms during a 15-min helicopter VR exposure. Peppermint- and ginger-flavored gums were equally effective, as compared to not chewing gum during the task. Moreover, we found a significant negative relationship between pleasant taste and VIMS, in the sense that the more pleasant the taste was perceived, the less severe VIMS symptoms were reported.

### Chewing gum to reduce VIMS

We described four possible mechanisms for the alleviating effect of chewing gum on VIMS (see Fig. 1): (1) mechanical noisy stimulation of (or interference with) the vestibular system, (2) physiological modulation by active ingredients, (3) distraction due to attention shift, and (4) emotional modulation induced by a pleasant taste. Considering that both kinds of chewing gum were equally effective in reducing VIMS and the taste was perceived as equally pleasant, we argue that the effect was more likely due to the vestibular stimulation from chewing and/or the positive emotions induced by the taste, rather than due to ginger as an active ingredient. Thus, mechanism (2) can be ruled out unless a believable physiological mechanism can be found for peppermint, which is on par with that postulated for ginger (see below). The observed effect of chewing gum on VIMS symptoms is in line with the findings of Darvall et al. (2017) who showed that chewing gum reduced postoperative nausea. In their study, they also used an ordinary peppermint gum without an additional active ingredient. A likely mechanism for the effect of chewing gum might be that chewing stimulated the vestibular system via mastoid vibration, which in turn reduces the visual–vestibular conflict by down-weighting the noisy afferent vestibular cues. We cannot say with certainty whether the mechanical process of chewing reduced symptoms, because we did not include a taste-neutral condition. However, it is a reasonable explanation, since previous studies have shown that stimulation of the mastoid through bone-conducted vibration (Weech et al. 2018) as well as vibrations administered through a headrest (Bos 2015) lead to a reduction in VIMS symptoms. Since seat vibration was found to be ineffective in reducing VIMS (D'Amour et al. 2017), vibration to the head appears to be essential for stimulating the vestibular system.

Since postural instability is considered to be a consequence of sensory conflict (see Bos 2011), the finding that gum chewing improves postural stability, as described by Alghadir et al. (2015), can also be considered in the context of sensory conflicts. Thus, gum chewing may improve postural stability by reducing visual–vestibular conflict, notwithstanding opposing views (e.g., Stoffregen and Riccio 1991).

This leaves mechanism (3) and (4) to consider. Another possible explanation for the positive effect of peppermint and ginger chewing gum on VIMS could be distraction through the motoric task of gum chewing. Since previous findings on mental distraction due to attention shift are contradictory, and chewing gum is not as mentally distracting as the audio letter memorizing task used in the study by Bos (2015), we tend to believe that an attention shift to the gum chewing action does not play a major role.

Mechanism (4), in contrast, needs to be looked at more closely. Chewing flavored gum could have served as a pleasant stimulus that evoked a positive mood. The underlying mechanism lies in modulating the negative emotion from the adverse stimulus into a more pleasant one, as discussed in studies that applied pleasant music, odors, or airflow as countermeasures (D'Amour et al. 2017; Keshavarz et al. 2015; Keshavarz and Hecht 2014; Peck et al. 2020). In our case, this could be caused by a pleasant taste or by pleasant memories of past experiences when chewing gum. Our finding that subjects reported less VIMS when the taste of the chewing gum was perceived as more pleasant further supports this assumption. Note that in the current data as well as in those reported by Keshavarz et al. (2015), the pleasantness of the stimuli only had an effect when it was consciously perceived, suggesting that mechanism (4) has a cognitive dimension.

When considering all possible mechanisms (see Fig. 1), we favor as likely mechanisms down-weighting due to mechanical stimulation induced by the chewing action (1) and emotional modulation (4), and possibly both in unison. Physiological modulation by active ingredients of ginger (2) and mere attention shift away from nausea symptoms (3) are less convincing.

### Natural remedy for VIMS

In our study, we found no additional beneficial effect of ginger on VIMS, as compared to the peppermint flavor. This is contradictory to the findings by Lien et al. (2003), who exposed 13 subjects to circular vection and discovered that administration of ginger powder reduced vasopressin plasma levels and nausea. They administered 1 or 2 g of ginger powder, which may or may not have been a larger dose than absorbed via the chewing gum we have used. As the manufacturer of the latter did not specify how much ginger it contained and the chewing gum was not produced for medical purposes, differences in the amount of active ingredient could explain the varying findings. Another major difference is the method of administration. Since medicated chewing gums were overall found to be effective in systemic release of active ingredients, easy-to-administer, and highly accepted (Kumari et al. 2020), we do not believe that administration via chewing gum explains the null-effect of ginger in our study. Compared to capsules, the rate of drug absorption via chewing gum is even faster and serum concentrations are similar in caffeine and dimenhydrinate chewing gums (Kamimori et al. 2002; Skofitsch and Lembeck 1983). However, contradictory and ambiguous results have been found for its anti-emetic effect on motion sickness. Palatty et al. (2013) attribute this to differences in the origin, time of harvest, and extraction method of ginger. Be this as it may, the role of ginger, as a countermeasure for VIMS remains unclear. Since the administration of ginger is low cost and does not result in any known adverse side-effects, ginger can be attempted individually as a remedy for VIMS.

### Practical implications

VR is an emerging technology that could be used in numerous important applications, such as in the training of emergency personnel or helicopter crews. The use of VR technologies offers the opportunity to train the entire crew together before entering into the expensive and resource-intense real helicopter training. Our results validate the use of chewing gum as an easy-to-use countermeasure against VIMS with great acceptance and entirely free of the side-effects associated with more potent medications, such as the drowsiness induced by dimenhydrinate.

We conducted a first study exploring the potential of chewing gum to mitigate VIMS in VR. Chewing gum is non-invasive, affordable, accepted, and easily accessible means to reduce VIMS. Most sickness using VR is caused by a visual–vestibular conflict, which is not fully eliminated by chewing gum. Thus, chewing gum is not potent enough to completely eliminate VIMS. The chewing gum groups did experience an increase in VIMS after all, but it contributes to the overall well-being of users. For instance, a chewing gum could be used for the first steps in VR to make the adaptation to nauseous stimuli more comfortable. It should be noted that the beneficial effects of chewing gum may or may not generalize to real-world cases of nausea in helicopter training, such as carsickness, which is not necessarily related to VIMS in simulators (see Bos et al. 2021).

### Limitations and future directions

In the present study, we highlighted the role of chewing gum in VIMS and its possible underlying mechanisms. We were able to rule out some of these mechanisms, however, the exact mechanism by which chewing gum alleviates VIMS symptoms remains unclear. In particular, as our study was not designed to distinguish between the mechanical effects of chewing and the flavor experience, it remains for future studies to investigate the effects of chewing and taste separately to further differentiate among the underlying mechanisms.

Due to necessary exclusions, the sample size was ultimately smaller than the number of recruited subjects, resulting in minor differences between the groups. The ginger group was slightly smaller and with less male subjects compared to the other groups. It should be noted, however, that the groups did not differ significantly with respect to the distribution of sex, age, or motion sickness susceptibility.^2^

The treatment-related differences between the groups were found to be significant for the FMS scores, but not for all SSQ subscales. Rather than questioning the effects obtained with the FMS data, we believe that the SSQ exhibits its maximum sensitivity only at high degrees of VIMS. At the more moderate levels obtained here, the FMS is more sensitive and thus the more appropriate measure for two reasons. First, the scale is firmly anchored at both ends with a range of response options from 0 to 20. The SSQ scores are more or less ordinal rankings. Second, we only have two SSQ scores pre- and post-exposure, whereas VIMS was assessed via FMS once a minute during exposure. It is thus not surprising that the FMS captured VIMS changes that were not detected by the SSQ. In contrast, the FMS cannot differentiate among symptom categories, and only the SSQ was able to reveal the importance of disorientation as main symptom.

Our simulation produced relatively mild VIMS symptoms (FMS mean peak for the control group: *M* = 4.56, SD = 3.52), although our pre-test with a small sample had shown stronger symptoms (FMS mean peak: *M* = 6.29, SD = 5.02). Thus, our results may apply only to the reduction of mild VIMS symptoms. In a simulation that triggers stronger VIMS, we would expect larger effects. However, it remains to be investigated if chewing gum has comparable effects on more severe VIMS. Replication studies with different applications, larger sample sizes, and different kinds of chewing gum would be highly desirable to confirm the detected effects. In particular, it would be interesting to have the subjects choose their favorite chewing gum to create even stronger pleasant distraction as Peck et al. (2020) did with self-selected music.

In our study, the association between taste perceived as pleasant and VIMS symptomatology suggests that emotional modulation has an influence on VIMS. As more studies point to such a direction, the direct influence of positive emotions should be further explored. Future research could also investigate a combination of chewing gum and other pleasant stimuli, such as music or airflow, to induce a stronger positive emotional state potentially reducing VIMS. Likewise, other promising natural remedies such as vitamin C should be further investigated.

### Conclusion

In the present study, we found that both a peppermint and a ginger-flavored chewing gum were able to significantly alleviate VIMS in a virtual helicopter flight, as compared with a control group that did not chew gum. In contrast to our hypothesis, the active ingredient in ginger did not show any additional beneficial effect. Moreover, we detected a significant negative relationship between the perceived pleasantness of taste and VIMS. Among the different mechanisms that might be responsible for the positive effects of chewing flavored gum on motion sickness, we regard mechanical vestibular stimulation via the chewing action and emotional modulation to be the most likely candidates. The former may cause the vestibular signal to receive less weight, the latter may involve experienced pleasantness. Further investigation of the underlying mechanisms of how chewing gum may reduce the visual–vestibular conflict will help to develop even better countermeasures for VIMS to improve user acceptance of VR technology in numerous application fields like education, training, or clinical settings.

## Supplementary Information

Below is the link to the electronic supplementary material.

Supplementary file1 (DOCX 13 KB)

## Footnotes

1 The non-rounded *p* value is less than .05 (*p* = .04982).

2 An additional multiple regression analysis including age, sex, and motion sickness susceptibility as control variables resulted in the identical pattern of results regarding group effects.
