# Supplementary material for: Chewing gum reduces visually induced motion sickness
Source: Exp Brain Res. 2022 Jan 7;240(2):651–63. doi: 10.1007/s00221-021-06303-5 (PMC8741140; doi:10.1007/s00221-021-06303-5)
Supplement: Supplementary file 1 — Supplementary file1 (DOCX 13 KB) [file 221_2021_6303_MOESM1_ESM.docx]

## Covid-19 Precaution Measures

Due to the Covid-19 pandemic during the trial period, a hygiene concept was developed involving precautionary measures to prevent infections. Before participating, subjects were kindly informed to stay at home in case they did not feel well or showed any Covid-19-related symptoms. When entering the room, the subjects disinfected their hands and wore a face mask. As soon as they sat down, they were allowed to take it off. Thus, bodily symptoms were not influenced by wearing a mask during the VR exposure and when completing the self-report questionnaires. The experimenter disinfected her hands each time she entered the room, wore a mask throughout the experiment, and maintained a safe distance of approximately three meters from the subject. Between the trials, we aired the room, changed the pads of the VR-HMD, and disinfected all surfaces and equipment. After the trial, subjects left their contact information in a separate list to track possible infections.
